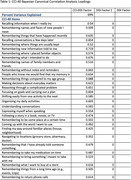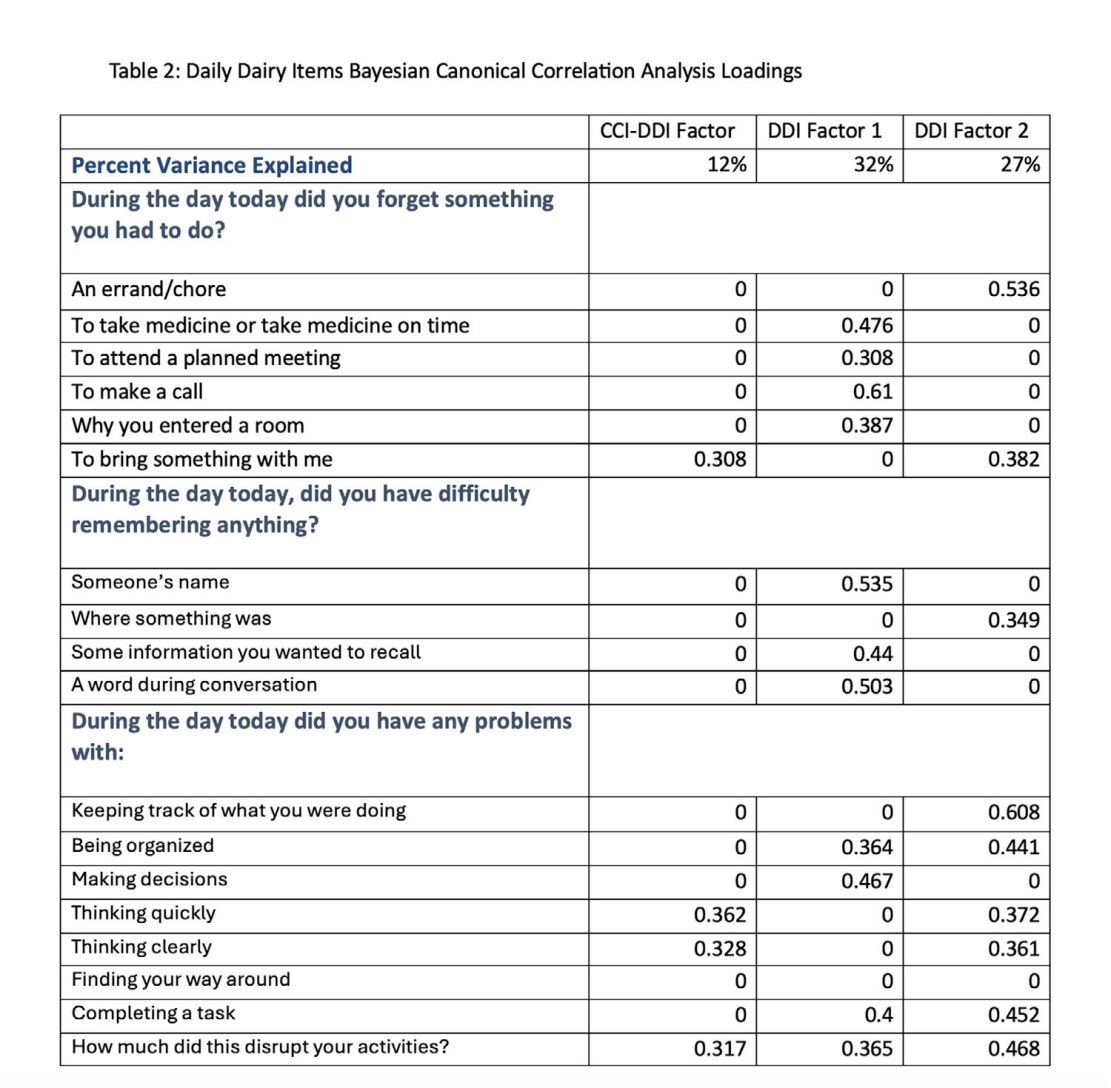# Optimizing the Assessment of Self‐Perceived Cognitive Functioning for Predicting Mild Cognitive Impairment: Integrating Traditional Methods with Daily Digital Diary Assessments: Results from the Einstein Aging Study (EAS)

**DOI:** 10.1002/alz.092072

**Published:** 2025-01-03

**Authors:** Angel Garcia De La Garza, Jacqueline A. Mogle, Cuiling Wang, Mindy J. Katz, Carol A. Derby, Jack Cameron, Richard B. Lipton, Laura Rabin, Martin J. Sliwinski

**Affiliations:** ^1^ Albert Einstein College of Medicine, Bronx, NY USA; ^2^ Clemson University, Clemson, SC USA; ^3^ Department of Neurology, and Department of Epidemiology and Population Health, Albert Einstein College of Medicine, Bronx, NY USA; ^4^ Brooklyn College of the City University of New York, Brooklyn, NY USA; ^5^ Pennsylvania State University, State College, PA USA

## Abstract

**Background:**

Ecological momentary assessments (EMA) are increasingly used to monitor self‐perceived memory and cognitive difficulties. We investigate how traditional self‐reported, recall based assessments of cognitive difficulties correlate with EMA measures. We identify factors explaining shared variance between measures from the 40‐item version of the Cognitive Change Index (CCI) and from EMA daily diaries, and factors explaining unique variance in each assessment. We compare EMA and CCI individually, as well as factors derived from both assessments, in predicting incident mild cognitive impairment (MCI).

**Methods:**

In neuropsychologically unimpaired Einstein Aging Study participants, we collected daily digital diary data on 18 cognitive lapses and their perceived disruption of everyday function over 14 days. Additionally, we assessed the conventional recall‐based indicators using the paper‐and‐pencil CCI, referencing both current ability and change over five years. Bayesian Canonical Correlation Analysis (BCCA) was employed to identify factors explaining the relationship between EMA and CCI, and factors explaining the unique variance in each. Separate Cox regressions were employed to assess whether baseline standardized CCI total scores, total number of cognitive lapses reported on EMA, and factors derived from the BCCA, were associated with the incidence of MCI after adjusting for age, gender, race/ethnicity, and depression status.

**Results:**

Analyses included 205 community‐dwelling participants (mean age = 77.11, SD = 4.78, 66.82% female, 53.17% white) without MCI at baseline. We identified one shared factor between CCI‐40 and EMA that explains 69% of CCI‐40 variability and 12% of EMA variability (canonical correlation = 0.58). We also found two factors unique to EMA explaining 32% and 27% of the variance. The shared CCI‐EMA factor (HR = 1.56, 95% CI = 1.11‐2.19, p = 0.0017), and total CCI‐40 scores (HR = 1.60, 95% CI = 1.14‐2.25, p = 0.011), significantly predicts MCI incidence at any follow‐up. Neither of total number of cognitive lapses reported on EMA nor BCCA factors unique to EMA significantly predict incidence MCI.

**Conclusions:**

Results suggest that traditional assessments and EMA measurements of memory and cognitive difficulties provide both shared and unique information. This information could be harnessed using advanced data analysis techniques to improve the prediction of incident MCI.